# Adiponectin deficiency is a critical factor contributing to cognitive dysfunction in obese mice after sevoflurane exposure

**DOI:** 10.1186/s10020-024-00954-0

**Published:** 2024-10-16

**Authors:** John Man Tak Chu, Suki Pak Wing Chiu, Jiaqi Wang, Raymond Chuen Chung Chang, Gordon Tin Chun Wong

**Affiliations:** 1 Department of Anaesthesiology, School of Clinical Medicine, LKS Faculty of Medicine, The University of Hong Kong, Room K424, Queen Mary Hospital, Pokfulam, Hong Kong, HKSAR China; 2 Laboratory of Neurodegenerative Disease, School of Biomedical Sciences, LKS Faculty of Medicine, The University of Hong Kong, L4-49, Laboratory Block, 21 Sassoon Road, Hong Kong, HKSAR China; 3 State Key Laboratory of Brain and Cognitive Sciences, The University of Hong Kong, Hong Kong, HKSAR China

**Keywords:** Obesity, Sevoflurane-induced neurotoxicity, Cognitive dysfunction, Neuroinflammation, Adiponectin

## Abstract

**Background:**

The number of major operations performed in obese patients is expected to increase given the growing prevalence of obesity. Obesity is a risk factor for a range of postoperative complications including perioperative neurocognitive disorders. However, the mechanisms underlying this vulnerability are not well defined. We hypothesize that obese subjects are more vulnerable to general anaesthesia induced neurotoxicity due to reduced levels of adiponectin. This hypothesis was tested using a murine surgical model in obese and adiponectin knockout mice exposed to the volatile anaesthetic agent sevoflurane.

**Methods:**

Obese mice were bred by subjecting C57BL/6 mice to a high fat diet. Cognitive function, neuroinflammatory responses and neuronal degeneration were assessed in both obese and lean mice following exposure to 2 h of sevoflurane to confirm sevoflurane-induced neurotoxicity. Thereafter, to confirm the role of adiponectin deficiency in, adiponectin knockout mice were established and exposed to the sevoflurane. Finally, the neuroprotective effects of adiponectin receptor agonist (AdipoRon) were examined.

**Results:**

Sevoflurane triggered significant cognitive dysfunction, neuroinflammatory responses and neuronal degeneration in the obese mice while no significant impact was observed in the lean mice. Similar cognitive dysfunction and neuronal degeneration were also observed in the adiponectin knockout mice after sevoflurane exposure. Administration of AdipoRon partially prevented the deleterious effects of sevoflurane in both obese and adiponectin knockout mice.

**Conclusions:**

Our findings demonstrate that obese mice are more susceptible to sevoflurane-induced neurotoxicity and cognitive impairment in which adiponectin deficiency is one of the underlying mechanisms. Treatment with adiponectin receptor agonist ameliorates this vulnerability. These findings may have therapeutic implications in reducing the incidence of anaesthesia related neurotoxicity in obese subjects.

**Supplementary Information:**

The online version contains supplementary material available at 10.1186/s10020-024-00954-0.

## Introduction

The post exposure effects of the volatile anaesthetic sevoflurane on the brain have been subjected to much debate. Sevoflurane can be neuroprotective in various circumstances, including cerebral ischaemia and reperfusion injury (Wen et al. 2016) and liposaccharide-induced neuroinflammation (Liu et al. 2021). However, it also exhibits neurotoxic effects in aged animals (Chai et al. 2022) or those with pre-existing cognitive impairment (Huang et al. 2022). Previously, sevoflurane was shown to induce neurotoxicity via increasing proinflammatory cytokines released by microglia (Smith et al. 2012; Li et al. 2017) and promoting tau protein activity (Huang et al. 2021). Interestingly, significant memory decline was also observed in diabetic rats following sevoflurane exposure (Li et al. 2017). To date, it remains inconclusive whether brains from obese individuals are more vulnerable to sevoflurane-induced neurotoxicity (SIN).

Adiponectin is a neuroprotective adipokine produced by adipose tissues and it regulates inflammatory and neurotrophic responses (Cypess 2022; Esfahani et al. 2015). Adiponectin deficiency is commonly observed in obese patients and in obese animal models (Nigro et al. 2014). Recent research reported that adiponectin deficiency impaired cognitive function through exacerbating neuroinflammation while treatment with adiponectin suppressed inflammatory cytokine release via AdipoR-AMPK signalling (Jian et al. 2019). Base on the neuroprotective and neurotrophic properties of adiponectin, we hypothesized that adiponectin deficiency places obese individuals at increased risk of SIN, manifesting as cognitive dysfunction (Wang et al. 2023). We first aim to evaluate the differences in susceptibility to SIN between in obese mice as compared to their lean counterparts. Thereafter we investigated the role of adiponectin deficiency in SIN by using an adiponectin knockout (APN-KO) mouse model with the same genetic background as the lean and obese mice. Our findings indicate that sevoflurane triggered neurotoxicity and cognitive impairment in the obese and APN-KO but not in lean mice. Supplementation with an adiponectin receptor agonist in both obese and APN-KO mice reduced SIN and cognitive dysfunction. Taken together, our findings support the hypothesis of adiponectin deficiency contributing to the SIN in obese subjects.

## Methods

### Animal groups

3-month-old male C57BL/6 mice were obtained from the Centre of Comparative Medicine Research, The University of Hong Kong. Animals were kept on a 12/12-hour light-dark cycle and had access to food and water ad libitum. All animal experimental protocols were approved by the Department of Health, HKSAR, China and Committee on the Use of Live Animals in Teaching and Research (CULATR, approval number: 5349-20), The University of Hong Kong. All animal houses and facilities are accredited by the AAALAC International. All animal protocols were carried out in Centre of Comparative Medicine Research and Department of Anaesthesiology, Laboratory Block, Sassoon Road, Hong Kong.

**Fig. 1:**
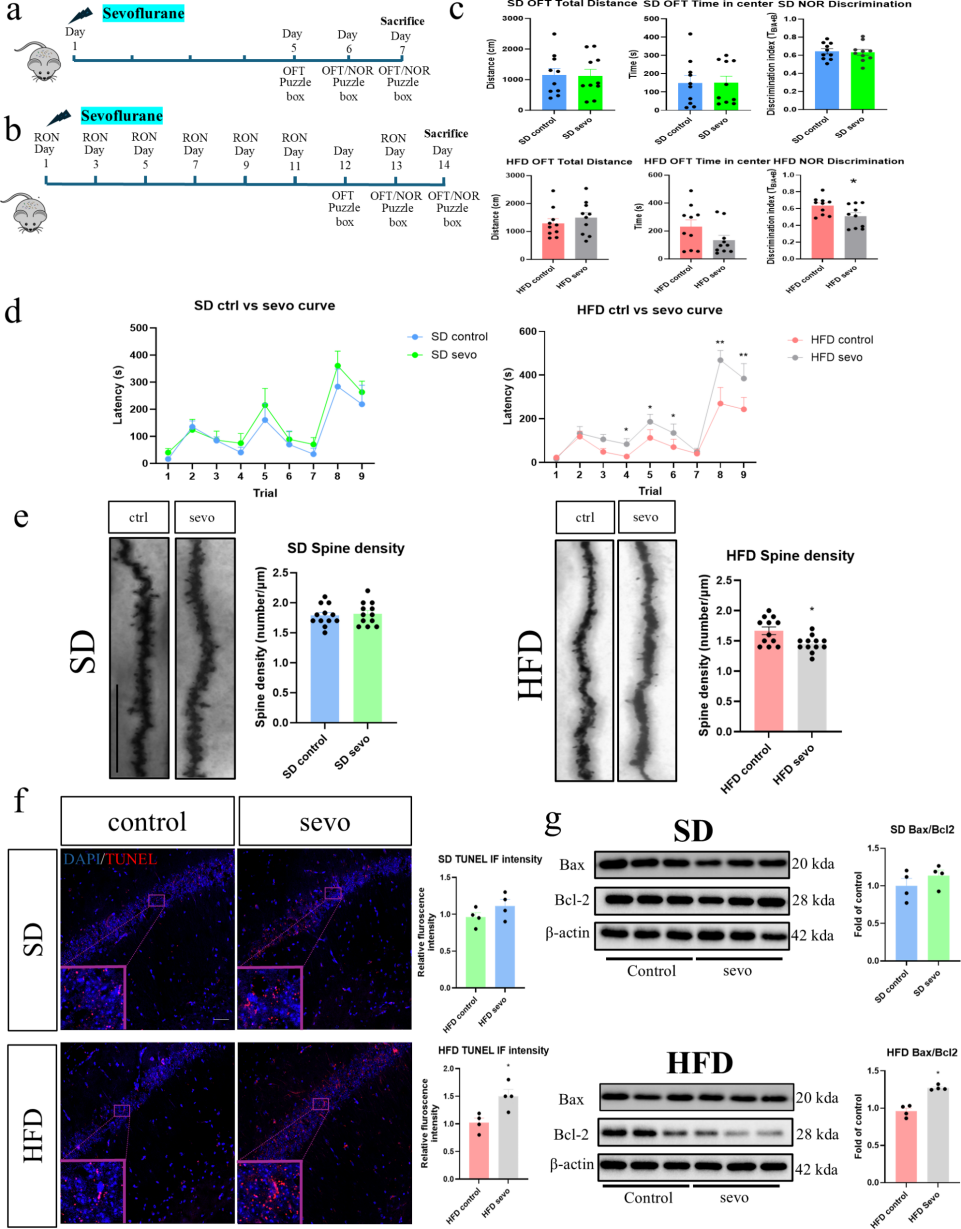
Neurotoxic effects of sevoflurane in obese mice. Sevoflurane induced cognitive dysfunction, dendritic spine loss and apoptosis in obese mice but not in lean mice. (**a** and **b**) The experimental timeline for sevoflurane exposure and AdipoRON treatment in obese and APN-KO mice. (**c**) Open field and NOR tests results in lean and obese mice. No significant changes in motor function or anxiety levels seen in any of the groups. A significant reduction in the discriminative index post exposure is seen in obese mice (*n* = 10). (**d**) Puzzle box test results in lean and obese mice. Significant increase in escape latency was observed in obese mice in different trials (*n* = 6). (**e**) Significant loss of dendritic spines was observed in obese mice after sevoflurane exposure. *n* = 12 in total, 3 dendrites were chosen at random from 4 mice in each group. (**f**) Up-regulation of TUNEL immunofluorescence (Scale bar = 50 μm) and (**g**) Bax/Bcl2 apoptotic signalling in obese mice after sevoflurane exposure (*n* = 4). All data are presented as the mean and SEM. Western blot data presented as the band densities normalized to β-actin. *p < 0.05, **p < 0.01 in comparison to respective control groups. Values were analysed using the unpaired t-test. SD = standard diet; HFD = high fat diet

Obese mice were bred from 3 months old C57BL/6 mice fed with a diet comprising of 60% fat content (D12492, Research Diet Inc, USA) for 3 months, whereas lean mice were fed with a standard diet. Body weights were monitored once per week. APN-KO mice with a C57BL/6 genetic background were kindly provided by Prof. Aimin Xu from Department of Medicine, The University of Hong Kong (Hui et al. 2015). Similarly aged 4-month-old male APN-KO mice were used in the experiments. The timeline for sevoflurane exposure, behavioural tests and animal sacrifice is shown in Fig. 1a.

### Sevoflurane exposure and AdipoRon treatment

Concentration of sevoflurane and the exposure duration were chosen according to our previous protocol (Huang et al. 2022). Briefly, mice were placed in an induction chamber filled with 5% of sevoflurane (SEVOrane, 100% w/w, Abbvie, USA) delivered in medical grade oxygen for 5 min (5% sevoflurane, 1 L/min airflow). Anaesthesia was then maintained for 2 h using 2.5% sevoflurane delivered at 1 L/min airflow while monitoring the respiratory rate. The mice were then allowed to emerge from anaesthesia and transferred to their home cages until their cognitive assessments.

AdipoRon is a selective agonist for the adiponectin receptor (Okada-Iwabu et al. 2013), and is dissolved with 10% of DMSO and 90% of corn oil (Sigma-Aldrich, USA) for oral administration. Obese and knockout mice were randomly pretreated with either 300 µl of vehicle or AdipoRon at a dose of 50 µg/kg/day (Ng et al. 2021) once every second day for 1 week. The animals were then exposed to sevoflurane as described above, with AdipoRon or vehicle continuing for another 7 days until the start of behavioural assessments. The timeline for AdipoRon treatment, sevoflurane exposure and animal sacrifice are shown in Fig. 1b. Animals were finally euthanized by CO_2_ asphyxiation followed by tissue harvesting.

### Open field and novel object recognition tests

Locomotor function and anxiety level of the animals were assessed by the open field test (OFT) (Huang et al. 2022). On day 5 post-exposure, mice were placed in the centre of an open arena box (40 cm x 40 cm x 40 cm) and were allowed to freely explore for 10 min. The length of distance travelled, and the centre duration time were then analysed using the SMART VIDEO TRACKING software (Harvard Apparatus, USA). On the video display, the base of the box was divided into 25 equal squares and centre duration time was defined as the length of the time that the mice stayed within the central 9 squares. A longer centre duration time represents a less anxious state of the mice.

Object recognition memory was determined by the novel object recognition (NOR) test as previously described (Huang et al. 2022). Briefly, on day 6 post-exposure, the mice were placed in the same box as in the open field test. Two identical objects were placed in the box and the mice were allowed to interact with the objects for 10 min. 24 h after this familiarization, one of the objects was replaced by a novel object. The mice were then allowed to interact with both objects for 10 min and their behaviour was analysed by the SMART VIDEO TRACKING software. The discrimination index is calculated using the formula of Tn/Tt, where Tn is the duration of time interacting with the novel object and Tt is the total time interacting with both the familiar and the novel object. A higher discrimination index indicates a better recognition memory in the test. Exclusion criteria included inadequate total object exploration time (less than 20s) or recognition bias towards a specific side of object during the familiarization period.

### Puzzle box test

The memory function and problem solving abilities were examined by the puzzle box test as described previously (Nada et al. 2011; Connor et al. 2014). The puzzle box consists of a brightly lit zone and a covered dark zone that are separated by a movable barrier. Mice were trained to move from the light zone into the dark zone through a narrow underpass located under the barrier. They underwent a total of nine trials (T1-T9), with three trials per day. Increasing difficulty of passage through the barrier was introduced day by day. Training began on day 5 post-exposure and the underpass was unobstructed during the first trial (T1). In T2 and T3, a channel was introduced through which the mice should enter the dark zone. The burrowing test was introduced on day 6. It began with T4 which was identical to T3. For T5 and T6, the underpass was filled with bedding that the mice needed to dig through to reach the dark zone. On day 7, the plug test was introduced with T7 being a repetition of T6 but for T8 and T9, a plug was placed and blocked the underpass that needed to be removed using their teeth and paws. Problem solving ability (T2, T5 and T8), short term memory (T3 and T6 and T9) and long-term memory (T4 and T7) were examined according to the sequence of the trials. A trial was started by placing the mouse in the light zone and ended when the mouse entered the dark zone or after a total time of 5 min (training and burrowing tests) or 10 min (plug test). After finishing each trial, each mouse were allowed to rest for 10 min before the next trial. The performance of animals was compared by measuring the escape latency in reaching the dark zone in each trial.

### TUNEL assay

DNA fragmentation in the hippocampus were examined using the TUNEL assay as previously described (Huang et al. 2022). Counterstaining of nuclei was performed by mounting the brain sections with prolong gold medium containing DAPI (Thermofisher Scientific, USA). Fluorescent images were obtained using a Carl Zeiss LSM780 laser scanning confocal microscope (ZEISS, Germany). Immunofluorescence intensities were analysed by ZEN software.

### Western blotting for protein analysis

Different phosphorylated and endogenous protein expression were examined by Western blotting. The hippocampal tissues were first homogenized in RIPA buffer supplemented with protease and phosphatase inhibitors (Roche, Switzerland). Protein samples were then extracted and resolved by SDS-PAGE, followed by transferring onto a PVDF membrane. The membrane was then incubated with primary and HRP-conjugated secondary antibodies. Protein signals were visualized with enhanced chemiluminescence reagents (Advansta Inc, USA) and captured by Chemidoc imaging system (Bio-Rad, USA). Quantification of the signals was performed by densitometric analysis of the Image Lab Software (Bio-Rad, USA).

### Immunofluorescence staining

Microglial and astrocytic activation in the hippocampus were examined by immunofluorescence staining. Briefly, brain tissues were harvested after perfusion with 0.9% saline and fixed with 4% paraformaldehyde solution for 24 h. 20 μm coronal sections containing the hippocampal region were prepared and mounted on the glass slide. Sections were first blocked by PBS with 1% BSA and 0.1% of Triton X reagent, then stained with the microglial marker IBA1 and the astrocyte marker GFAP antibodies for overnight. Sections were then incubated with a secondary antibody conjugated with Alexa Fluor dyes for 2 h. After incubation, sections were mounted with prolong gold medium containing DAPI (Thermofisher Scientific, USA). Fluorescent images were captured by the confocal microscope and the immunofluorescence intensities were analysed by ZEN software.

### Golgi staining for the number of dendritic spines

Dendritic spines in the hippocampus were examined by Hito Golgi-Cox OptimStain™ kit as previously described (Chu et al. 2022). Briefly, brain tissues were harvested without perfusion and rinsed with distilled water. Impregnation with Golgi stain was performed according to the manufacturer’s protocol. 150 μm coronal brain sections of the hippocampal region were taken using a cryostat. Dendritic spines on hippocampal neurons were then examined and their number counted under a light microscope, with the results expressed as the number of spines per µm of dendrite.

### ELISA assay and multiplex cytokine assays

ELISA and multiplex assays were used to examine adiponectin and inflammatory cytokines in the serum and hippocampus. Hippocampal tissues were harvested and extracted in lysis buffer supplemented with a protease inhibitor. The level of adiponectin in the serum and hippocampus was examined by ELISA according to the manufacturer’s protocol. Inflammatory cytokine expression in the hippocampus was examined by MILLIPLEX MAP mouse cytokine/chemokine magnetic bead panels (Merck Millipore, USA) according to the manufacturer’s protocol. Data were collected using a Bio-Plex 200 system (Bio-Rad, USA).

### Statistical analyses

Normality of the data were assessed using the Shapiro–Wilk normality test. The sample size to detect a significance for behavioural experiments and biochemistry studies is based on our previous studies (Huang et al. 2018, 2022). Data were analysed using an unpaired two-tailed t test or one way ANOVA with post-hoc test of Tukey. Data were presented as the mean ± standard error of the mean (SEM). Statistical significance was considered when *p* ≤ 0.05.

## Results

### Sevoflurane induced cognitive dysfunction in obese but not in lean mice

We first confirmed an adequate obese model with significant increase in body weight in mice following 12 weeks of high fat diet (Supplementary figure; Fig. S1). No significant differences in the total distance travelled and centre duration in the open field test were observed in both lean and obese mice post exposure (Fig. 1c), suggesting that sevoflurane did not exert any residual effects on locomotion and anxiety. On the other hand, a significant reduction in the discrimination index measured by the NOR was observed in the obese mice but not in lean mice, indicating that sevoflurane triggered a decline in object recognition memory (Fig. 1b). Results from the puzzle box test further indicated that sevoflurane adversely affected memory (T4, T6 and T9) and problem-solving abilities (T5 and T9) in the obese mice which was reflected by the significant increase of escape latency in the exposure group (Fig. 1c). On the contrary, no significant changes were found in the lean control groups (Fig. 1d). These results confirm an increased susceptibility in obese mice to the adverse cognitive effects of sevoflurane exposure but not in lean control.

### Sevoflurane induced the loss of dendritic spines and triggered apoptosis in the hippocampus of obese and not in lean mice

The number of dendritic spines on hippocampal neurons was assessed by Golgi staining and apoptosis was examined by TUNEL staining. In the lean groups, no significant differences in dendritic spines were observed while a significant decrease was found in obese mice after sevoflurane exposure (Fig. 1e). Similarly, no significant increases in TUNEL positive signals and pro-apoptotic Bax/Bcl-2 ratio was obeserved in lean groups (Fig. 1e and f). Conversely, a significant increase in TUNEL positive signals and Bax/Bcl-2 ratio were evident in the obese mice after sevoflurane exposure (Fig. 1f and g). These results demonstrated that sevoflurane induced synaptic loss and neuronal apoptosis in the hippocampus of obese mice while having minimum impact in lean mice.

### Sevoflurane induced neuroinflammatory changes in the hippocampus of obese mice and not in lean mice

**Fig. 2:**
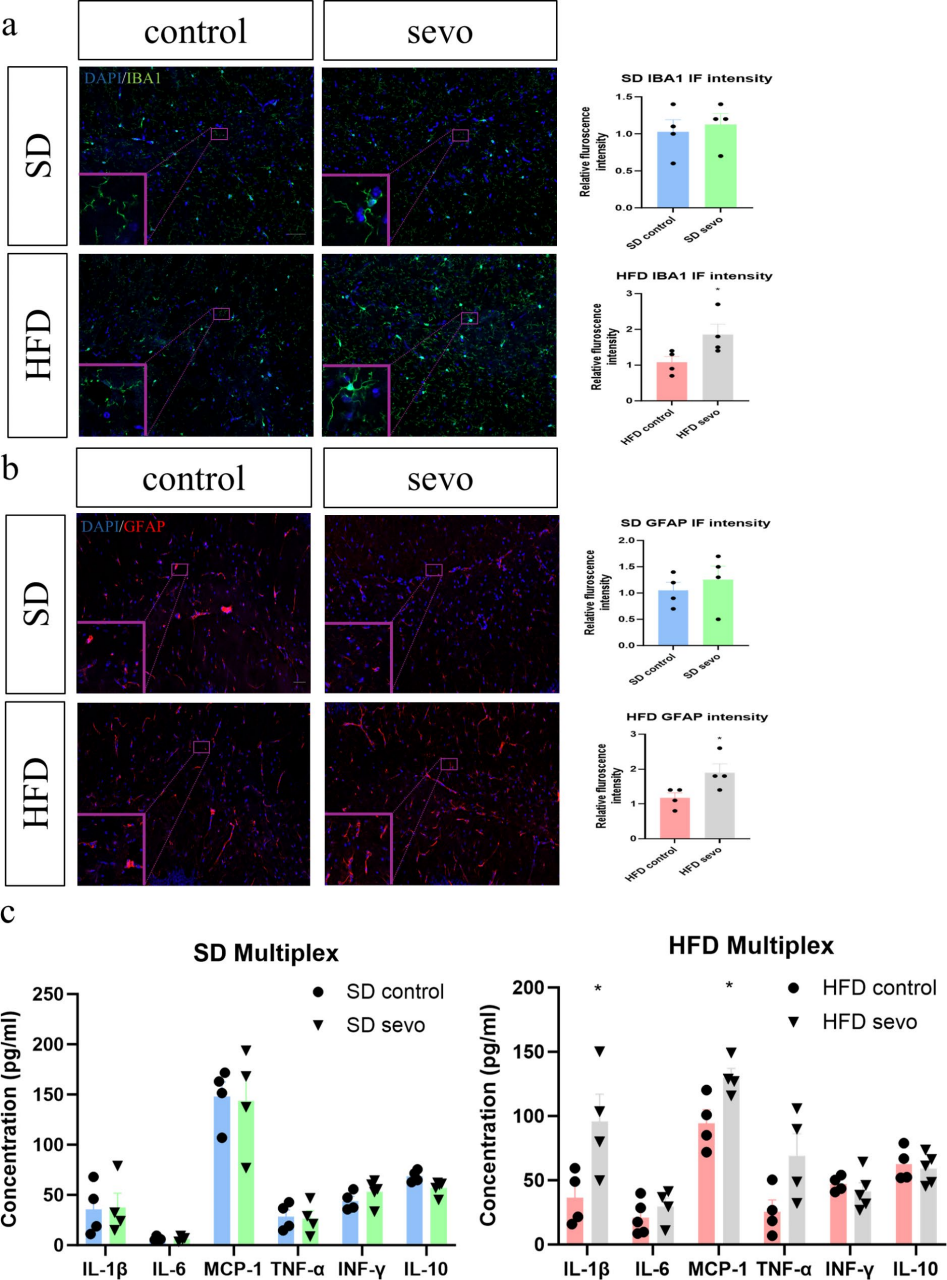
Neuroinflammatory responses in obese mice after sevoflurane exposure. (**a** and **b**) Significant increase in IBA1 and GFAP immunofluorescence in the hippocampus (Scale bar = 50 μm) in the obese group (*n* = 4). (**c**) Increases in protein expression of pro-inflammatory markers IL-1β and MCP-1 in the obese group (*n* = 4). All data are presented as the mean and SEM. *p < 0.05 in comparison to the respective control groups. Values were analysed using the unpaired t-test. SD = standard diet; HFD = high fat diet; IF = immunofluorescence

In obese mice, significant increases in IBA1 and GFAP immunofluorescence signals in hippocampus were observed whereas no significant changes were found in lean mice after sevoflurane exposure (Fig. 2a and b). From the multiplex assay, significant increases in inflammatory cytokines IL-1β and MCP-1 were found in the obese groups while no significant differences were observed between the lean groups (Fig. 2c). These data reveal that sevoflurane specifically triggers neuroinflammatory responses in the brains of obese but not lean mice.

### Sevoflurane modulated the AMPK/JNK pathway activity and increased neuropathological changes in the hippocampus of obese mice

**Fig. 3:**
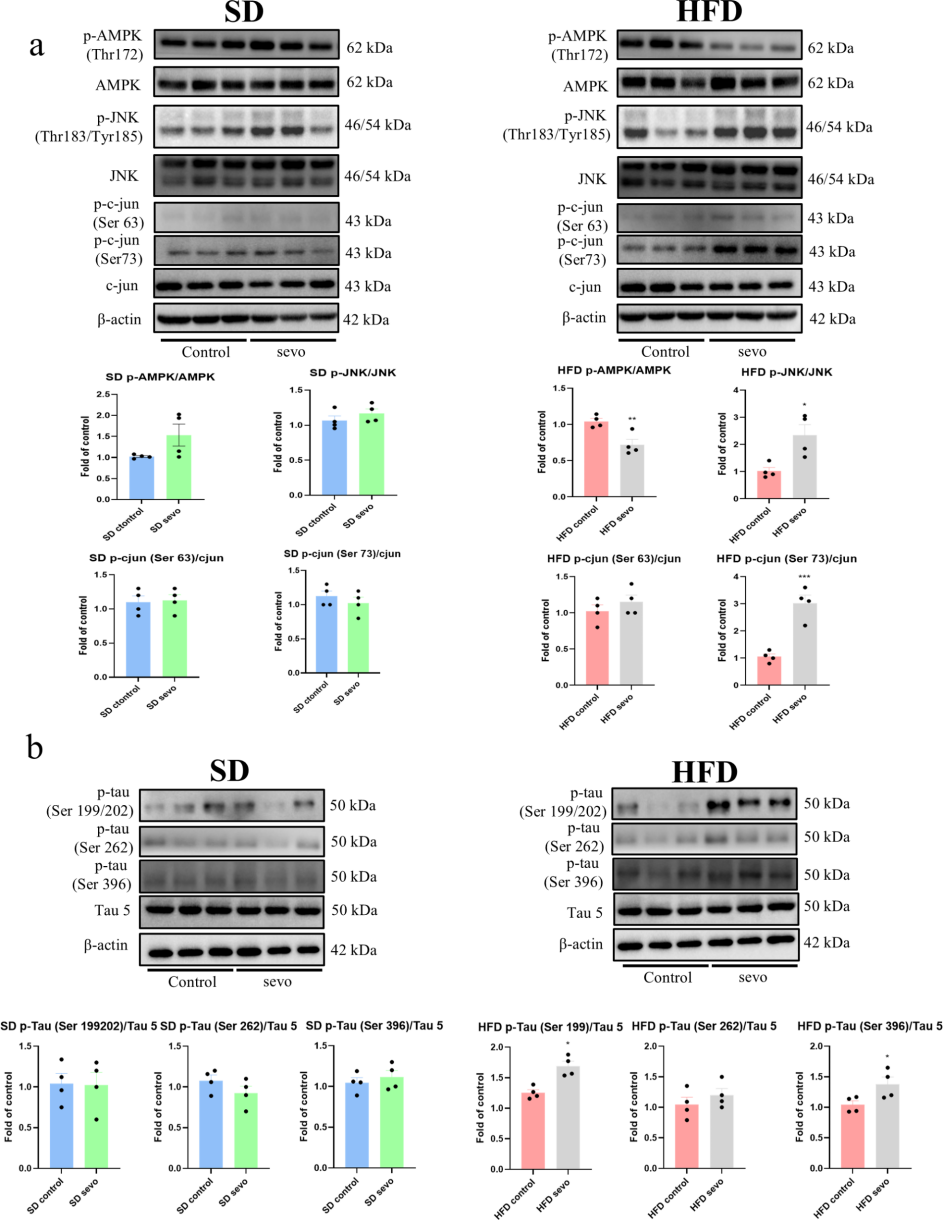
Activation of pro-inflammatory pathways and increased tau phosphorylation in obese mice after sevoflurane exposure. (**a**) Significant reduction in pAMPK and up-regulation of pJNK and c-jun (Ser 73) in the obese group (*n* = 4). (**b**) Increased tau phosphorylation (Ser 199 and 396) in the obese group (*n* = 4). All data are presented as the mean and SEM and presented as band densities normalized to their endogenous protein control. *p < 0.05, **p < 0.01, ***p < 0.001 in comparison to the respective control groups. Values were analysed using the unpaired t-test

Adiponectin binds to the adiponectin receptor and can inhibit neuroinflammatory responses through the AMPK pathway (Wang et al. 2023). We first demonstrated a significant reduction of adiponectin in the serum and hippocampus of obese mice (Supplementary figure; Fig. S2a) without the modulation of adiponectin receptor 1 among all animal groups (Supplementary figure; Fig. S2b). A significant reduction in phosphorylated AMPK was noted in the obese mice after post exposure (Fig. 3a). Besides, a significant up-regulation in phosphorylated JNK and c-jun, the downstream transcriptional factor of JNK, were also observed in obese groups (Fig. 3a).

As neuroinflammation is shown to correlate with different hallmarks of neuropathology such as tauopathy (Chen and Yu 2023), tau phosphorylation was also assessed by Western blot. Consistent with the above findings, a significant increase in phosphorylated tau at the Ser 199/202 and Ser 396 epitopes were observed in the obese mice (Fig. 3b). These findings imply that sevoflurane exacerbates neuroinflammatory responses in obese mice that correlates with the development of neuropathological changes.

### Sevoflurane induced cognitive impairment, neuroinflammatory responses, tau phosphorylation, apoptosis and dendritic spine loss and modulated the AMPK/JNK pathway activities in APN-KO mice

To confirm if adiponectin deficiency renders animals more susceptible to SIN, we repeated the above investigations in APN-KO mice fed with a standard diet. First, no significant weight differences were observed between wild type and APN-KO mice at similar ages (Supplementary figure; Fig. S3). Given that there were minimal cognitive and neuropathological impact of sevoflurane on lean mice as observed above, in the interest of reduction in animal use, we decided to omit this group from subsequent experiments.

**Fig. 4:**
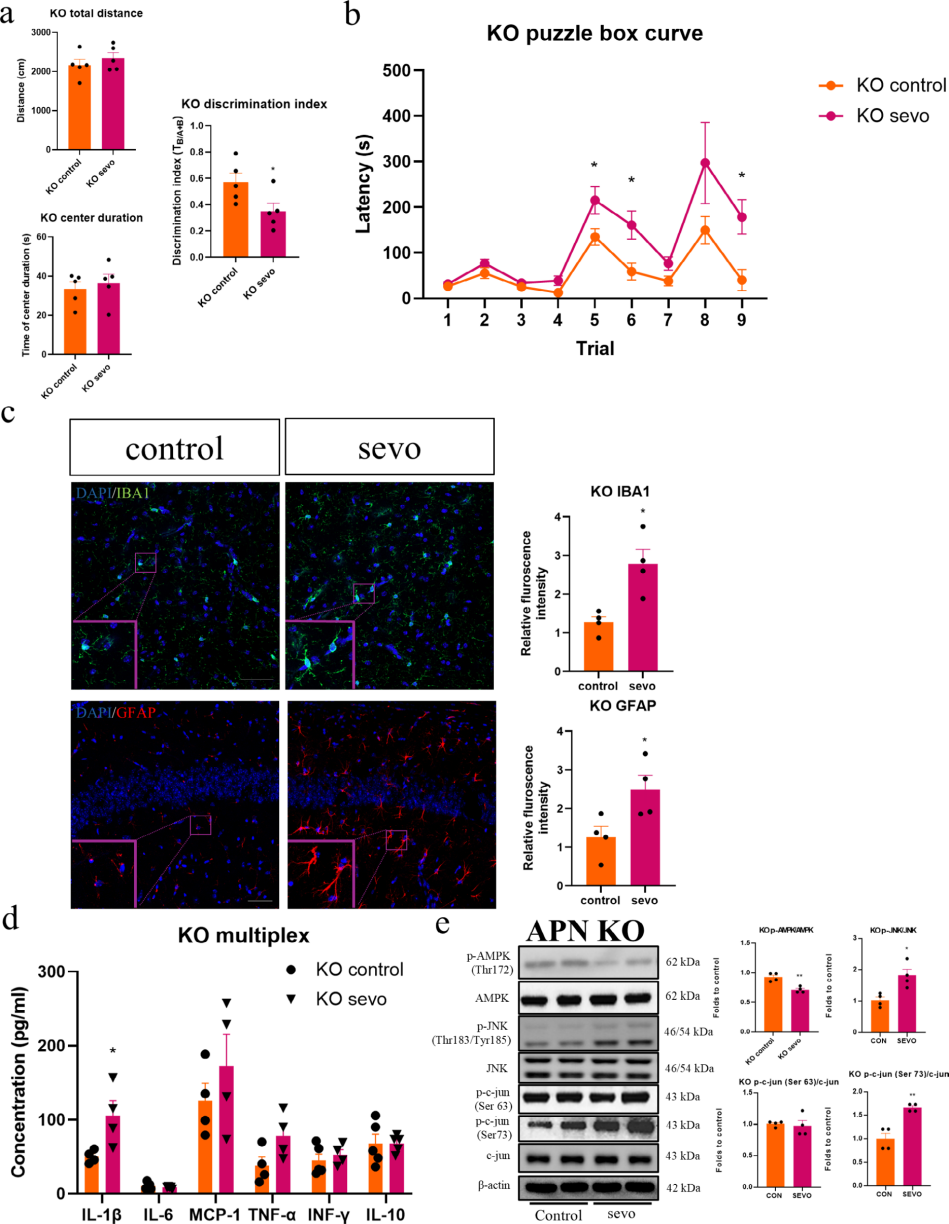
Cognitive decline and neuroinflammation in APN-KO mice after sevoflurane exposure. (**a** and **b**) No significant differences in locomotor activity or anxiety levels in APN-KO mice. Evidence for cognitive decline from NOR and puzzle box tests in adiponectin knockout mice (*n* = 4–5). (**c**) Significant increase in IBA1 and GFAP immunofluorescence in APN-KO mice (*n* = 4). Scale bar = 50 μm. (**d**) Multiplex assay indicated the increase in hippocampal IL-1b (*n* = 4). (**e**) Significant reduction in p-AMPK and increase in p-JNK and p-c-jun in APN-KO mice after sevoflurane exposure (*n* = 4). All data are presented as the mean and SEM. Western blot results represent the band densities that were normalized with endogenous β-actin. *p < 0.05, **p < 0.01 in comparison to the respective control groups. Values were analysed using the unpaired t-test. KO = adiponectin knockout

**Fig. 5:**
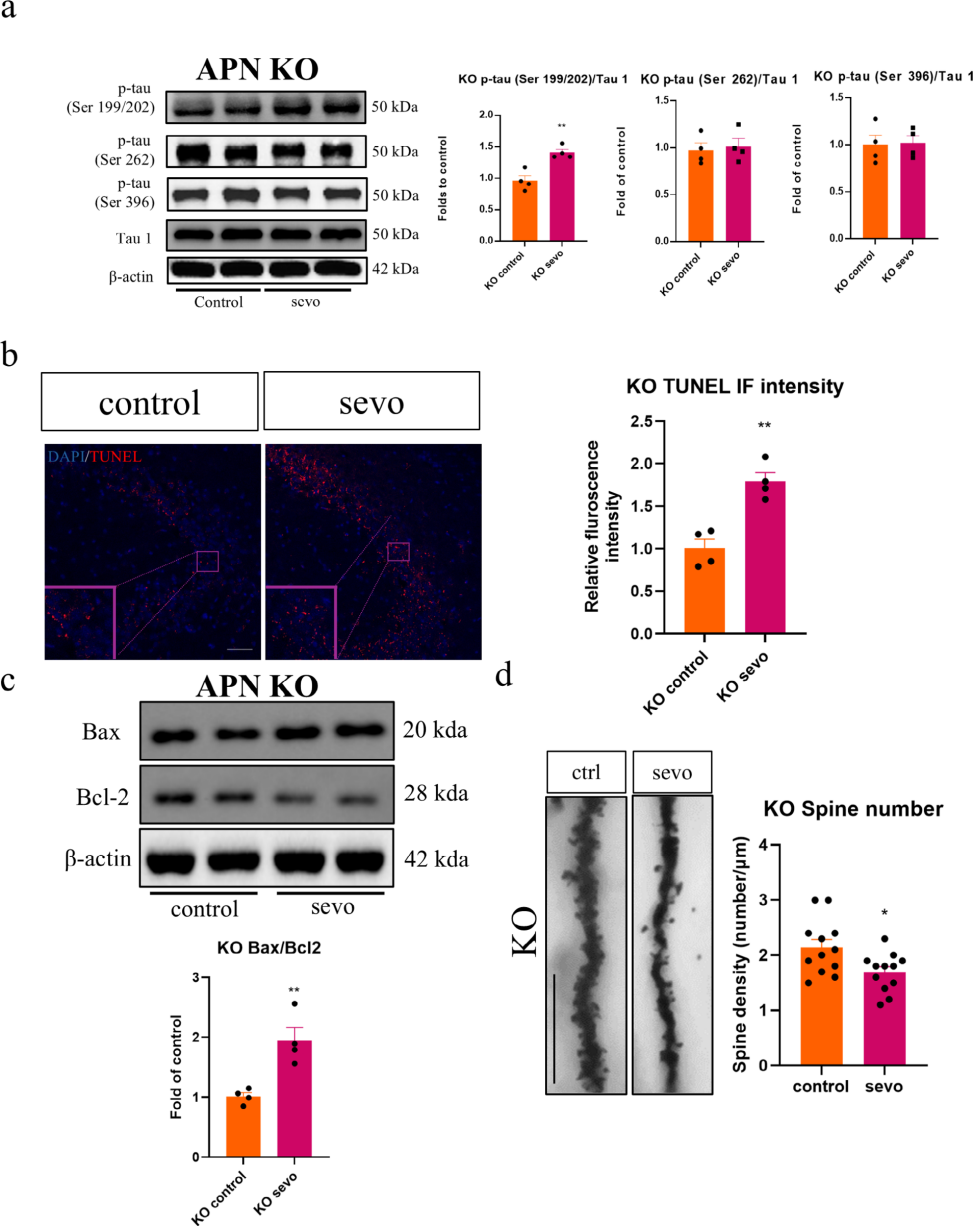
Tau phosphorylation, synaptic loss and apoptosis in APN KO mice after sevoflurane exposure. (**a**) Western blot results of phosphorylated tau in APN-KO mice after sevoflurane exposure. Significant up-regulation in p-tau (Ser 199/202) (*n* = 4). (**b**) Sevoflurane enhanced TUNEL immunofluorescence and (**c**) increased Bax/Bcl2 ratio in the hippocampus of APN-KO mice. Scale bar = 50 μm. *n* = 4. (**d**) Dendritic spine loss in APN- KO mice. *n* = 12 in total, 3 dendrites were chosen at random from 4 mice in each group. All data are presented as the mean and SEM. Western blot results represent the band densities that were normalized with their endogenous protein control. *p < 0.05, **p < 0.01 in comparison to the respective control groups. Values were analysed using the unpaired t-test

While no significant changes in locomotor activity or anxiety level were observed after sevoflurane exposure (Fig. 4a), the discrimination index was noted to be significantly in the APN-KO mice, which was in alignment with the results seen in the obese animals. Similarly, the puzzle box test also revealed a longer escape latency in both memory (T6 and T9) and problem-solving tasks (T5) in the post exposure group (Fig. 4b). In line with the observed cognitive decline, significant increases in IBA1 and GFAP immunofluorescent intensity, as well as IL-1β expression were also observed in the APN-KO mice (Fig. 4c and d). Consistent with the findings in obese mice, a significant reduction in phosphorylated AMPK, an increase in the expression of phosphorylated JNK, c-jun and phosphorylated tau in Ser 199/202 were seen (Figs. 4e and 5a). In terms of apoptosis, an increase in TUNEL immunofluorescence (Fig. 5b), coupled with an increased Bax/Bcl2 ratio (Fig. 5c) in the APN-KO mice post sevoflurane exposure. Finally, reduced dendritic spines was also observed in the APN-KO mice (Fig. 5d). These data from APN-KO mice strongly support the hypothesis that adiponectin deficiency plays a critical part in SIN and cognitive decline.

### Supplementation of AdipoRon improved sevoflurane-induced cognitive decline and neuropathology in obese and APN-KO mice

**Fig. 6:**
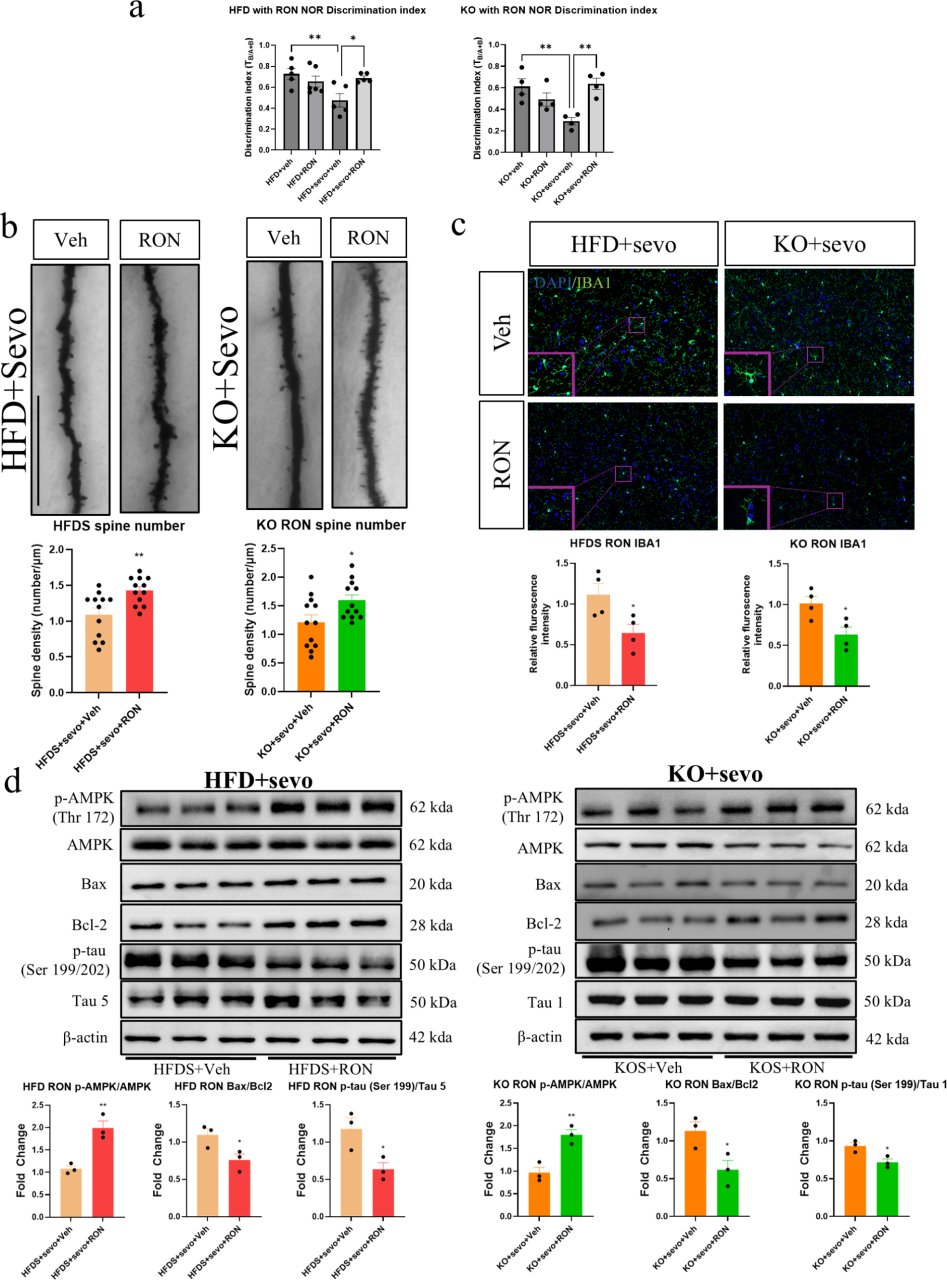
AdipoRon attenuated sevoflurane-induced neurotoxicity and cognitive dysfunction in both obese and APN-KO mice. (**a**) AdipoRon treatment attenuated sevoflurane-induced cognitive changes in both obese and APN-KO mice; (**b**) Reduced dendritic spine loss in AdipoRon treated groups (*n* = 12 in total, 3 dendrites were chosen at random from 4 mice in each group); (**c**) reduced microglial activation (Scale bar = 50 μm, *n* = 4) in AdipoRon treated groups; (**d**) AdipoRon treatment enhanced AMPK phosphorylation and inhibit Bax/Bcl2 apoptotic signalling and tau phosphorylation (Ser 199/202) in obese and KO mice (*n* = 3). All data are presented as the mean and SEM. Western blot data represent the band densities that were normalized with their endogenous protein control. *p < 0.05, **p < 0.01 in comparison to the respective control groups. Values were analysed using the unpaired t-test. HFDS = high fat diet with sevoflurane exposure; KOS = adiponectin knockout with sevoflurane exposure; Veh = vehicle solution; RON = AdipoRon

To examine whether administration of adiponectin receptor agonist can confer protection against SIN, AdipoRon was given to both obese and APN-KO mice starting 7 days before and continued for another 7 days after sevoflurane exposure. NOR test indicated a significant increase in the discrimination index after AdipoRon supplementation in both type of mice (Fig. 6a). Moreover, a significant increase of dendritic spines (Fig. 6b) and amelioration of microglial activation (Fig. 6c) were observed in the AdipoRon plus sevoflurane groups. From the Western blot analysis, a significant up-regulation of phosphorylated AMPK, together with the down-regulation of phosphorylated tau (Ser/199/202) and Bax/Bcl2 ratio were seen in both AdipoRon treated groups (Fig. 6d). These data suggest that AdipoRon can partially reverse the deleterious effects of sevoflurane in obese and APN-KO mice which may have translational value in the clinical setting.

## Discussion

Consistent with previous findings regarding obesity and cognition, we demonstrated that obese mice, having a lower level of adiponectin, had a greater tendency for cognitive dysfunction following sevoflurane exposure compared with their lean counterparts. This decrement in cognitive performance was accompanied by neuroinflammation, increased apoptosis, reduction in dendritic spines and increased tau phosphorylation. Normally both astrocytes and microglia play essential roles in supporting neuronal development and plasticity within the CNS by controlling metabolic and neurotrophic functions (Smith et al. 2012; Ding et al. 2021). However, when glial cells are activated as shown in our obese and APN-KO mice following sevoflurane exposure, they can also induce a neuroinflammatory state by releasing chemokines and inflammatory cytokines including IL-1β and TNF-α (Ding et al. 2021). These neuroinflammatory changes are implicated in cognitive dysfunction and correlate with the activation of astroglia and cytokines accompanying with neuropathological changes in other forms of dementia such as Alzheimer’s disease (AD) (Kinney et al. 2018).

In addition to demonstrating astroglial activation and increased inflammatory cytokines, we also investigated the downstream modulation of the intracellular AMPK/JNK pathways following sevoflurane exposure. Activation of JNK leads to phosphorylation of transcription factors, such as c-jun, which stimulates the expression of pro-inflammatory genes (Yan et al. 2024). Conversely, AMPK plays a key role in inhibiting inflammatory responses by influencing JNK and NF-κB signalling (Xiang et al. 2019; Chen et al. 2018a, b). Neuroinflammatory conditions such as diabetes or AD have been shown to cause AMPK inhibition alongside JNK and NF-κB pathway upregulation (Chen et al. 2018a, b; Peixoto et al. 2017). Our data demonstrated a significant reduction of phosphorylated AMPK in obese animals after sevoflurane exposure, associated with increased phosphorylated JNK and c-Jun, however no significant corresponding changes were observed in lean mice.

A significant loss of dendritic spine and an increase in apoptotic signals in hippocampus of obese were observed following sevoflurane exposure (Fig. 1). The synapse stands as a critical locus of neurodegeneration preceding the onset of cognitive deficits (Selkoe 2002; Chi et al. 2018) and the degree of synaptic loss and neuronal apoptosis correlate with the extent of cognitive decline (Selkoe 2002; Chi et al. 2018). In terms of apoptosis, opposing effects of sevoflurane have been observed in in vivo animal models. In some of the animal disease models, sevoflurane could attenuate the neuronal apoptosis (Shi et al. 2020; He et al. 2018; Bedirli et al. 2018). Nevertheless, pro-apoptotic effects of sevoflurane have also reported in the animal models of neonatal, pre-existing cognitive impairment and metabolic disorders (Huang et al. 2022; Li et al. 2020; Han et al. 2021). These observations are in line with our current findings that with the pathological background of metabolic disorders, prolonged sevoflurane exposure may trigger neuronal apoptosis and hence lead to synaptic degeneration. In apoptotic cells, DNA fragmentation and the activation of the pro-apoptotic Bax/Bcl2 pathway are characteristically seen (Czabotar and Garcia-Saez 2023). Bax is a key component in initiating apoptosis by increasing mitochondrial permeability, which initiating apoptosis (Czabotar and Garcia-Saez 2023), while anti-apoptotic Bcl2 controls mitochondrial permeabilization, thus inhibiting the downstream apoptotic pathway and facilitating cell survival (Czabotar and Garcia-Saez 2023). Substantial evidence has indicated the involvement of neuronal apoptosis in other cognitive dysfunction models such as Alzheimer’s disease and vascular dementia (Wang et al. 2020; Wojcik et al. 2024). In those disease models, apoptosis will result in the synaptic degeneration and the subsequent neuronal loss in the memory circuit which may lead to cognitive impairment. Our current findings indicated an increase in the Bax/Bcl2 ratio and the TUNEL signals in both obese and APN-KO mice after sevoflurane exposure, supporting the potential involvement of apoptosis in the observed SIN.

Phosphorylation of tau and the subsequent tauopathy are thought to be associated with the progression of neuroinflammation (Chen and Yu 2023). Phosphorylation of tau results in its dissociation from axonal microtubules and form oligomers, which cause neurotoxicity and subsequent synaptic dysfunction (Barbier et al. 2019). Under neuroinflammatory conditions, JNK acts as a MAP kinase and regulate tau phosphorylation on different amino acid residues, including serine 199, 202, 396, and 404 (Wang and Mandelkow 2016). Moreover, inflammatory cytokines including IL-1β can promote tau phosphorylation through various signalling pathways (Ghosh et al. 2013). Previous reports described sevoflurane induced tau phosphorylation in postnatal mice resulting in later cognitive dysfunction (Tao et al. 2014; Liang et al. 2023). Consistent with these findings, our data also revealed a significant increase of tau phosphorylation in obese mice, but not in lean mice.

Whether one develops post operative cognitive decline is dependent on multiple factors. Patients with major risk factors for developing perioperative neurocognitive disorders (PNDs) such as advanced age or pre-existing cognitive impairment, are more prone to a neuroinflammatory state. This pro-inflammatory tendency may result in a greater neuroinflammatory response in the brain when further triggered. Neuroinflammation is an early event involved in the neurotoxicity of sevoflurane in other models (Huang et al. 2022; Neag et al. 2020). Major surgical trauma triggers an acute systemic inflammation and a significant number of cases can lead to a neuroinflammatory response that could disrupt cognitive processes.

Therefore, the presence of cognitive deficits in the absence of surgery in this study is a rather significant finding. The impact of general anaesthesia alone on the brain remains a topic of debate, as both neuroprotective (Wen et al. 2016; Wang et al. 2022) and detrimental effects (Chai et al. 2022; Huang et al. 2022; Zuo et al. 2020) have been reported. It has been proposed that such differential effects depend on the state of neuronal development and pathological background (Neag et al. 2020). With regards to the CNS, obesity is associated with decreased grey matter volume, accumulation of neuropathology and neuroinflammatory response (Jiang et al. 2023; Miller and Spencer 2014; Tabassum et al. 2020). Recently obesity has been identified as an independent risk factor for post operative cognitive dysfunction (Burns et al. 2023). Furthermore, in both clinical observations and animal studies, subjects with metabolic syndrome have a higher risk in developing different postoperative complications, including experiencing cognitive decline (Feng et al. 2013; Tzimas et al. 2015). These findings imply that the brain from an obese individual is already under “stressed” condition which may make it more vulnerable to the SIN.

Adiponectin is one of the most abundant adipokine possessing anti-inflammatory properties, as well as regulating energy expenditure via lipid and glucose metabolism (Bloemer et al. 2018; Kadowaki and Yamauchi 2005). Circulating adiponectin can pass through the blood brain barrier and bind to adiponectin receptors, thereby regulate cerebral energy homeostasis, hippocampal neurogenesis and synaptic plasticity (Bloemer et al. 2018). Apart from obese patients, a reduced circulating adiponectin level is also observed in patients with mild cognitive impairment and AD (Teixeira et al. 2013). Chronic adiponectin deficiency is associated with the accumulation of AD related neuropathological changes and cognitive deficits in aged animals (Ng et al. 2016). In contrast, treatment with adiponectin can enhance insulin sensitizing effects and improve cognitive dysfunction in AD animals (Ng et al. 2021). Adiponectin can exert its neurotrophic and neuroprotective effects via the binding to the adiponectin receptor 1 and 2 (AdipoR1 and AdipoR2) (Bloemer et al. 2018). Binding of adiponectin to AdipoR can enhance the association of AdipoR1 with APPL1, subsequently results in the binding of APPL1 with AMPKα2, leading to AMPK phosphorylation and activation (Fang et al. 2010). AMPK activation significantly attenuate the JNK-NF-κB signalling cascade and inhibited mRNA and protein levels of pro-inflammatory cytokines (Chen et al. 2018a, b). In the CNS, phosphorylation of AMPK induces an anti-neuroinflammatory response by inhibiting the JNK pathway in a mouse model of intracerebral haemorrhage (Chen et al. 2018a, b). These reports indicate the important role of adiponectin in cognitive dysfunction and anti-neuroinflammatory response. Adiponectin deficiency may render the brain more vulnerable to exogenous insults. Our hypothesize that obese subjects are more susceptible to the neurotoxicity of sevoflurane owing to a deficiency of adiponectin in subjects with excess adiposity seem to be supported by our results.

To confirm if adiponectin deficiency is indeed a key factor contributing to SIN in vulnerable groups, APN-KO mice, fed with a standard diet to reduce the chance of developing obesity, were similarly exposed to sevoflurane. Similar neurotoxic effects and cognitive deficits were observed in these mice, which included cognitive impairment, neuroinflammatory responses, inhibition of AMPK, phosphorylated JNK and c-jun and increased tau phosphorylation at residue 199, apoptosis and dendritic spine loss. These data from APN-KO mice confirm that pre-existing of deficiency of adiponectin, but not necessarily adiposity, is sufficient to induce cognitive decline, neuroinflammatory response and neuronal degeneration by sevoflurane exposure (Figs. 4 and 5). This further support our hypothesis that obese mice are more susceptible to the neurotoxic effects of sevoflurane due to adiponectin deficiency.

To further confirm the key role of adiponectin, we proceed to evaluate whether augmenting the biological actions of adiponectin in both obese and APN-KO mice would negate the adverse effects from sevoflurane. We used the synthetic selective adiponectin receptor agonist AdipoRon to mimic the actions of adiponectin. This orally active agent circumvents some limitations of converting the native protein into a viable pharmacological agent, such as the diverse range of protein structure expressed and the insolubility of the C-terminal domain (Barbalho et al. 2023). It can cross the blood brain barrier and its use have been shown to ameliorate Alzheimer like neuropathological traits in AD animal models (Ng et al. 2021; Khandelwal et al. 2022). We demonstrated that supplementation of AdipoRon attenuated sevoflurane-induced microglial activation, synaptic loss, and cognitive decline in both obese and knockout mice, which are consistent with previous studies and confirm the critical role of adiponectin deficiency in SIN.

Though our data is compelling for a protective effect of adiponectin against the neurotoxic effects of sevoflurane in the obese mice, our experimental design has a few limitations which need to be highlighted. First, few patients would clinically be exposed to anaesthesia in the absence of surgery, so we did not use a true representative model of PNDs. We examined the protective effect of only one adiponectin substitute that has been previously studied experimentally while others are also available. We administered AdipoRon for some time across the peri-exposure period but did not assess the cognitive behaviour just prior to the animal being subjected to anaesthesia so we might have already altered the animal’s baseline with the supplement. Finally, the beneficial effects of AdipoRon may not be limited to obese animals and it would be interesting to investigate in other models of PNDs.

## Conclusions

In conclusion, the brains of obese subjects exposed to sevoflurane maybe more vulnerable to develop adverse cerebral effects in part due to the relative lack of neurotrophic effects from adiponectin. Supplementation with the adiponectin substitute AdipoRon mitigated against SIN. Given the scope of the global obesity epidemic and the demand for surgical procedures in this population, perioperative neurocognitive disorders may place an increasing burden to health systems worldwide and adversely affected patients’ post operative quality of life. As adiponectin substitutes are being actively investigated for a range of obesity related metabolic disease, our findings provide an impetus to examine the role of these agents in cerebral protection during the perioperative period for these patients.

## Electronic supplementary material

Below is the link to the electronic supplementary material.

Supplementary Material 1

Supplementary Material 2

Supplementary Material 3

Supplementary Material 4

Supplementary Material 5

## Data Availability

The datasets used and/or analyzed during the current study are available from the corresponding author on reasonable request.

## Abbreviations

AD: Alzheimer’s disease
APN-KO: Adiponectin knockout
CNS: Central nervous system
GFAP: Glial fibrillary acidic protein
IBA1: Ionized calcium binding adaptor molecule 1
NOR: Novel object recognition
SIN: Sevoflurane-induced neurotoxicity
TUNEL: TdT-mediated dUTP nick-end labelling
