# Supplementary figures and images for: Adiponectin deficiency is a critical factor contributing to cognitive dysfunction in obese mice after sevoflurane exposure
Source: Mol Med. 2024 Oct 16;30:177. doi: 10.1186/s10020-024-00954-0 (PMC11481458; doi:10.1186/s10020-024-00954-0)

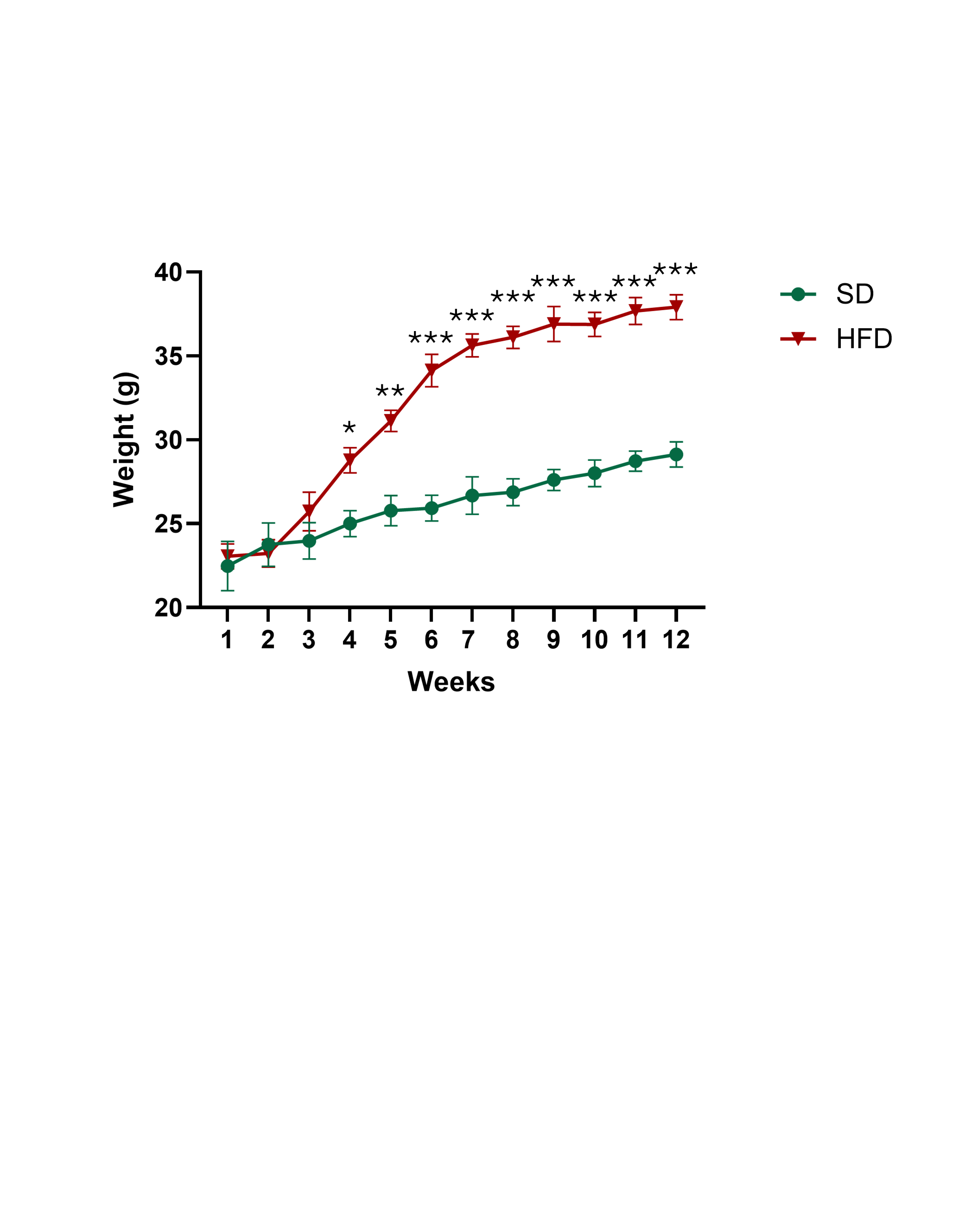

Supplement: Supplementary file 1 — Supplementary Material 1 [file 10020_2024_954_MOESM1_ESM.tif]

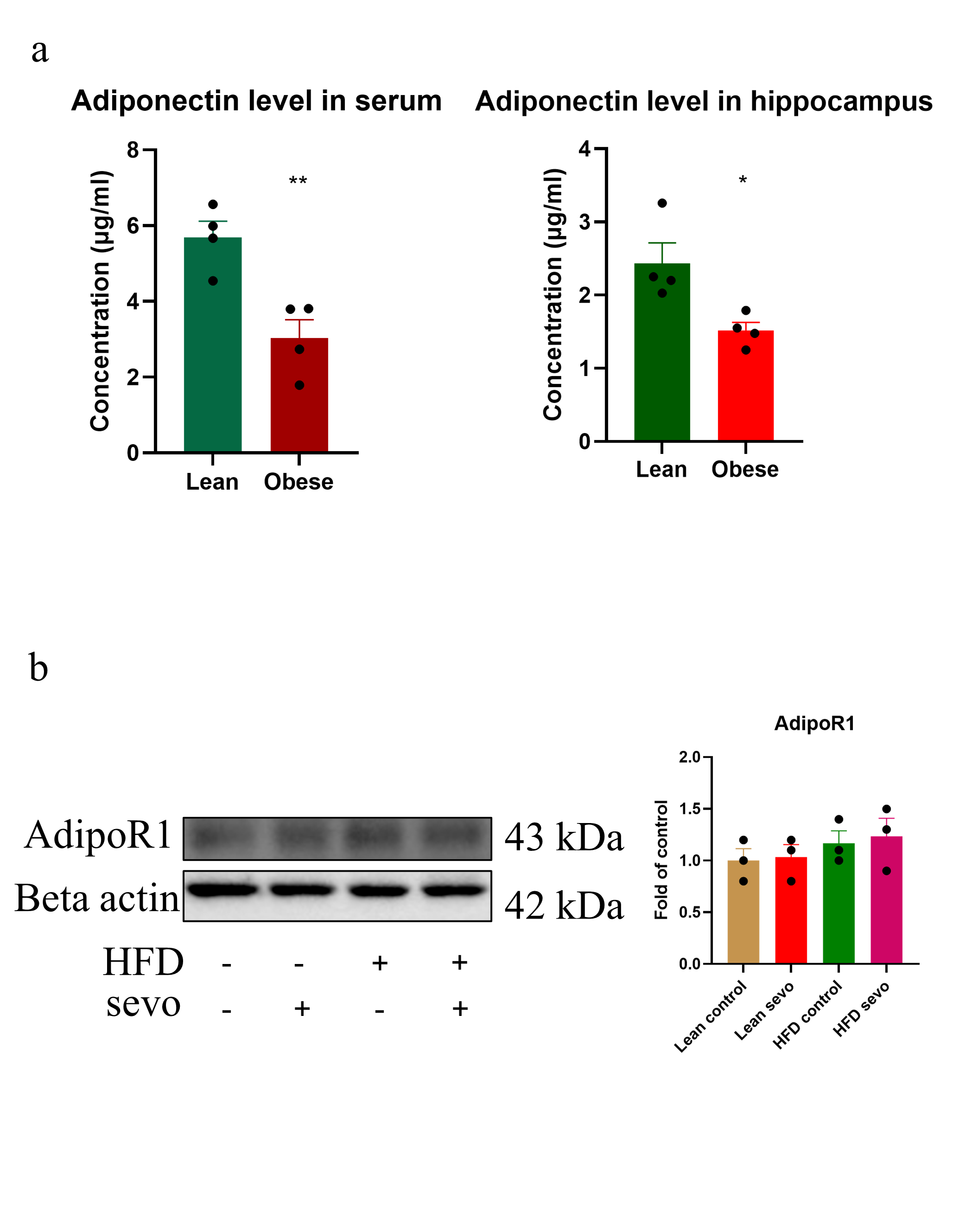

Supplement: Supplementary file 2 — Supplementary Material 2 [file 10020_2024_954_MOESM2_ESM.tif]

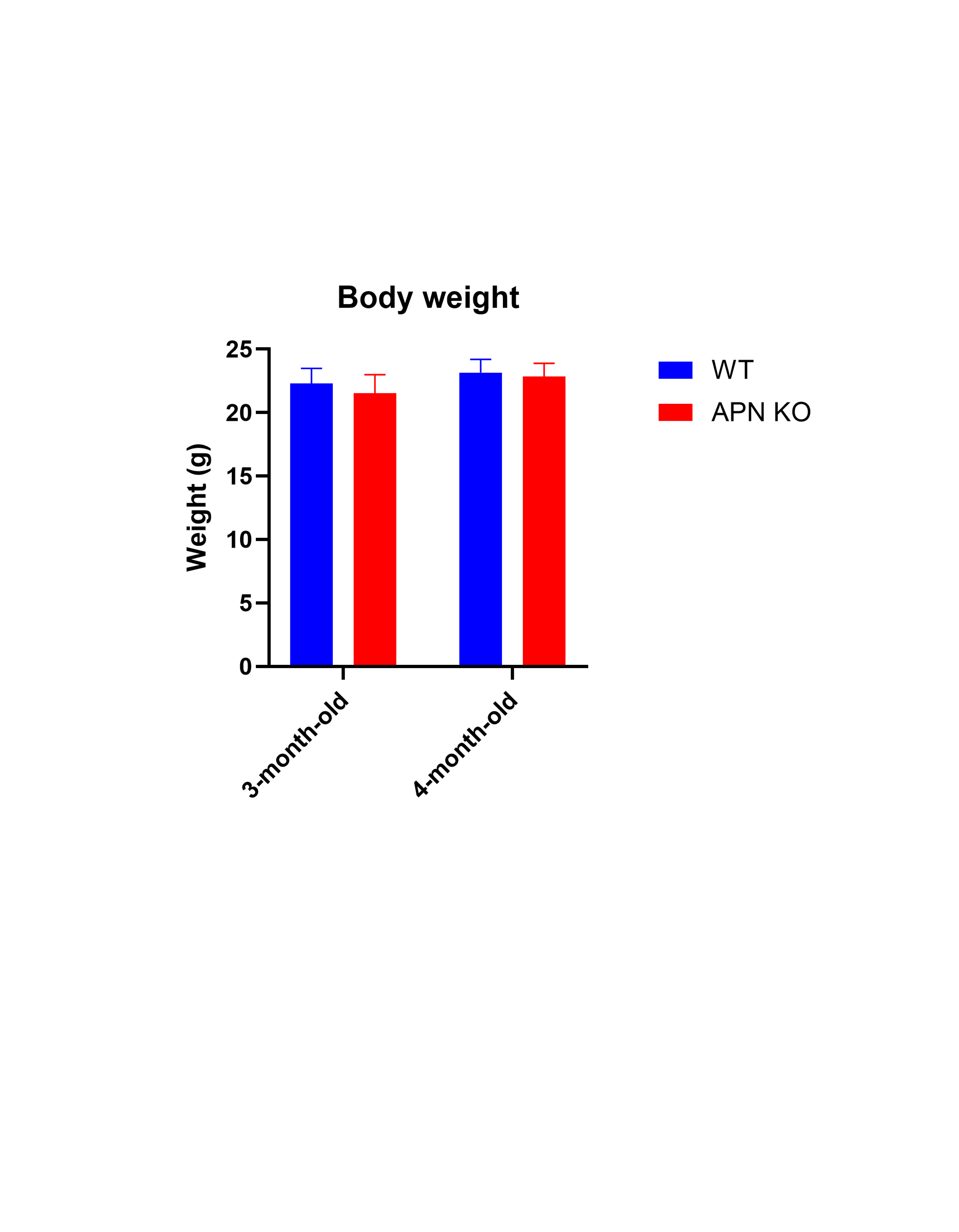

Supplement: Supplementary file 3 — Supplementary Material 3 [file 10020_2024_954_MOESM3_ESM.tif]
