# Supplementary material for: Adiponectin deficiency is a critical factor contributing to cognitive dysfunction in obese mice after sevoflurane exposure
Source: Mol Med. 2024 Oct 16;30:177. doi: 10.1186/s10020-024-00954-0 (PMC11481458; doi:10.1186/s10020-024-00954-0)
Supplement: Supplementary file 4 — Supplementary Material 4 [file 10020_2024_954_MOESM4_ESM.docx]

| Antibodies | Supplier | Cat number | Antibody dilution | Activation or inhibition |
| --- | --- | --- | --- | --- |
| Bax | Cell Signaling | 2772 | 1:1000 | NA |
| Bcl-2 | Cell Signaling | 3498 | 1:1000 | NA |
| β-actin | Sigma-Aldrich | A2228 | 1:20000 | NA |
| GFAP | Sigma-aldrich | G3893 | 1:2000 | NA |
| IBA1 | Fujifilm | 019-19741 | 1:2000 | NA |
| p-AMPK | Cell Signaling | 2535 | 1:2000 | Activate |
| AMPK | Cell Signaling | 5831 | 1:3000 | NA |
| p-JNK | Cell Signaling | 9255 | 1:2000 | Activate |
| JNK | Cell Signaling | 9252 | 1:2000 | NA |
| p-c-jun (Ser 63) | Cell Signaling | 2361 | 1:1000 | Activate |
| p-c-jun (Ser 73) | Cell Signaling | 9164 | 1:1000 | Activate |
| c-jun | Cell Signaling | 9165 | 1:2000 | NA |
| p-tau (Ser 199/202) | Thermofisher Scientific | 44-768G | 1:2000 | Activate |
| p-tau (Ser 262) | Thermofisher Scientific | 44-750G | 1:2000 | Activate |
| p-tau (Ser 396) | Thermofisher Scientific | 44-752G | 1:2000 | Activate |
| Tau5 | Thermofisher Scientific | MA5-12808 | 1:3000 | NA |
| Tau1 | Sigma-Aldrich | MAB3420 | 1:3000 | NA |
| AdipoR1 | Abcam | ab70362 | 1:1000 | NA |
|  |  |  |  |  |
| Assay Kit and reagents |  |  |  |  |
| Adiponectin ELISA kit | R&D system | MRP300 |  |  |
| In Situ Cell Death Detection Kit | Roche | 11684795910 |  |  |
| Hito Golgi-Cox OptimStain™ Kit | Hitobiotech | HTKNS1125 |  |  |
| MILLIPLEX MAP mouse cytokine/chemokine magnetic bead panels | MilliporeSigma | MCYTOMAG-70K |  |  |
| AdipoRON | MedChemExpress | HY-15848 |  |  |
| High fat diet | Research Diets | D102010 |  |  |
