# Supplementary material for: Adiponectin deficiency is a critical factor contributing to cognitive dysfunction in obese mice after sevoflurane exposure
Source: Mol Med. 2024 Oct 16;30:177. doi: 10.1186/s10020-024-00954-0 (PMC11481458; doi:10.1186/s10020-024-00954-0)

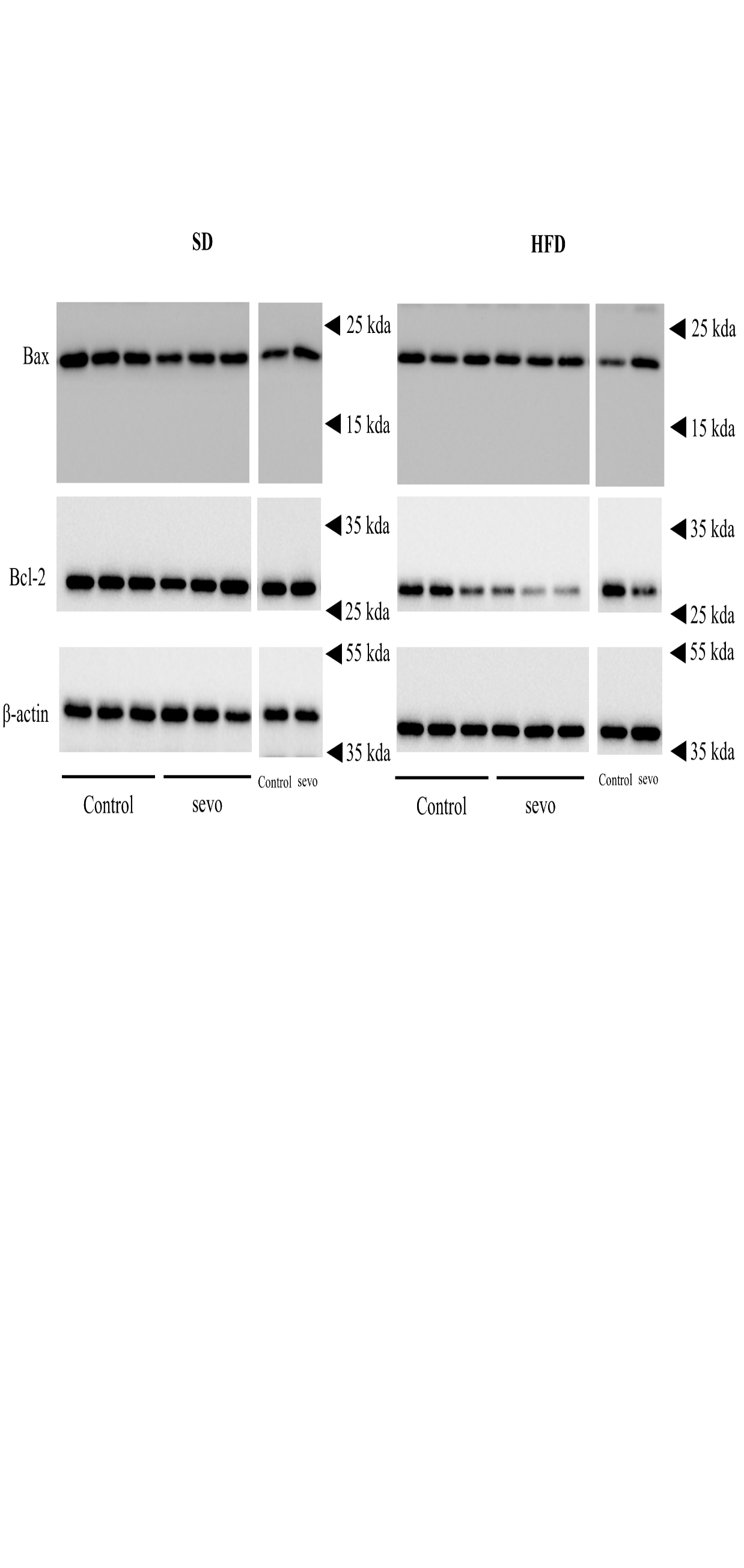
Fig. 1 Western blot


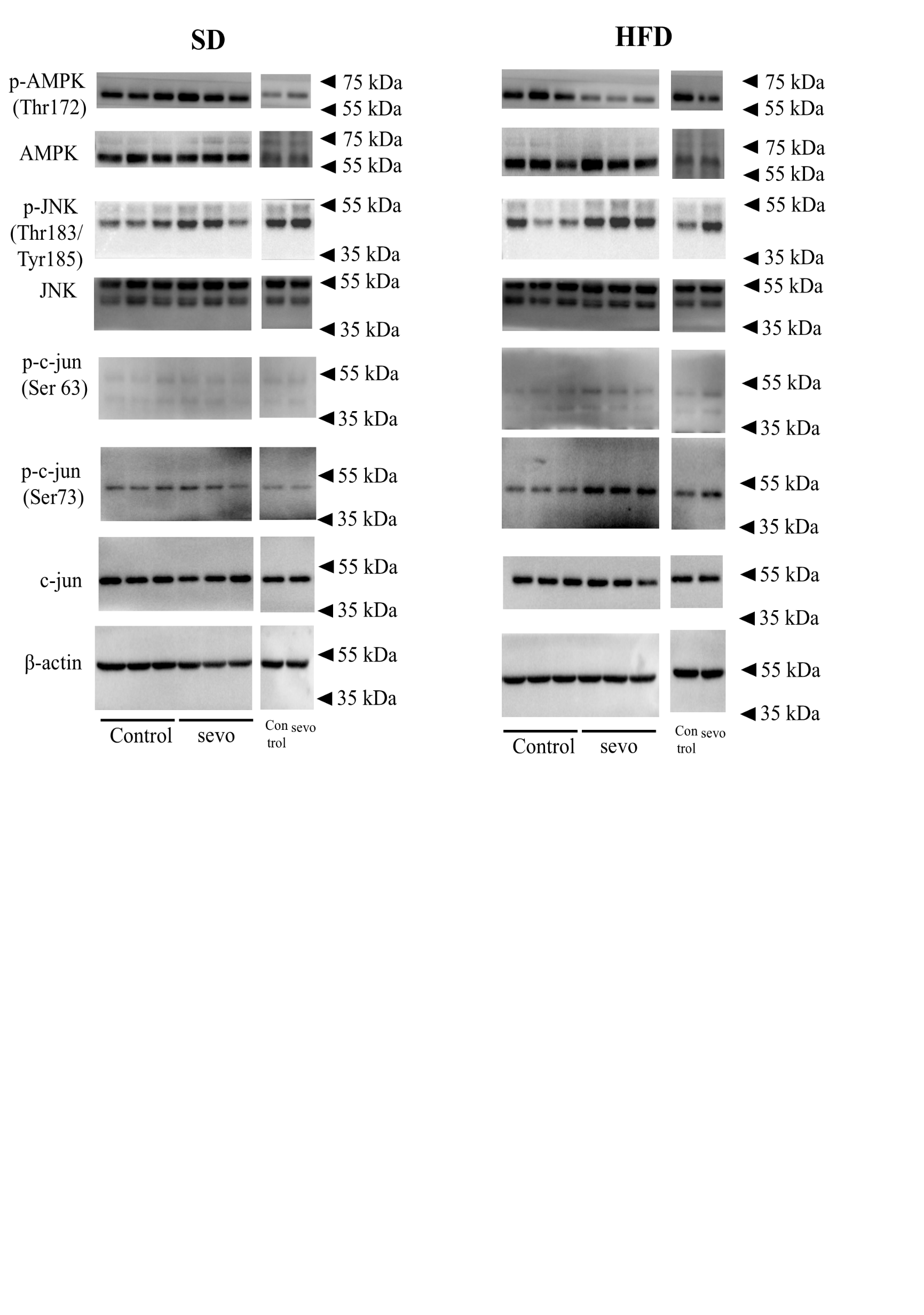
Fig. 3a Western blot


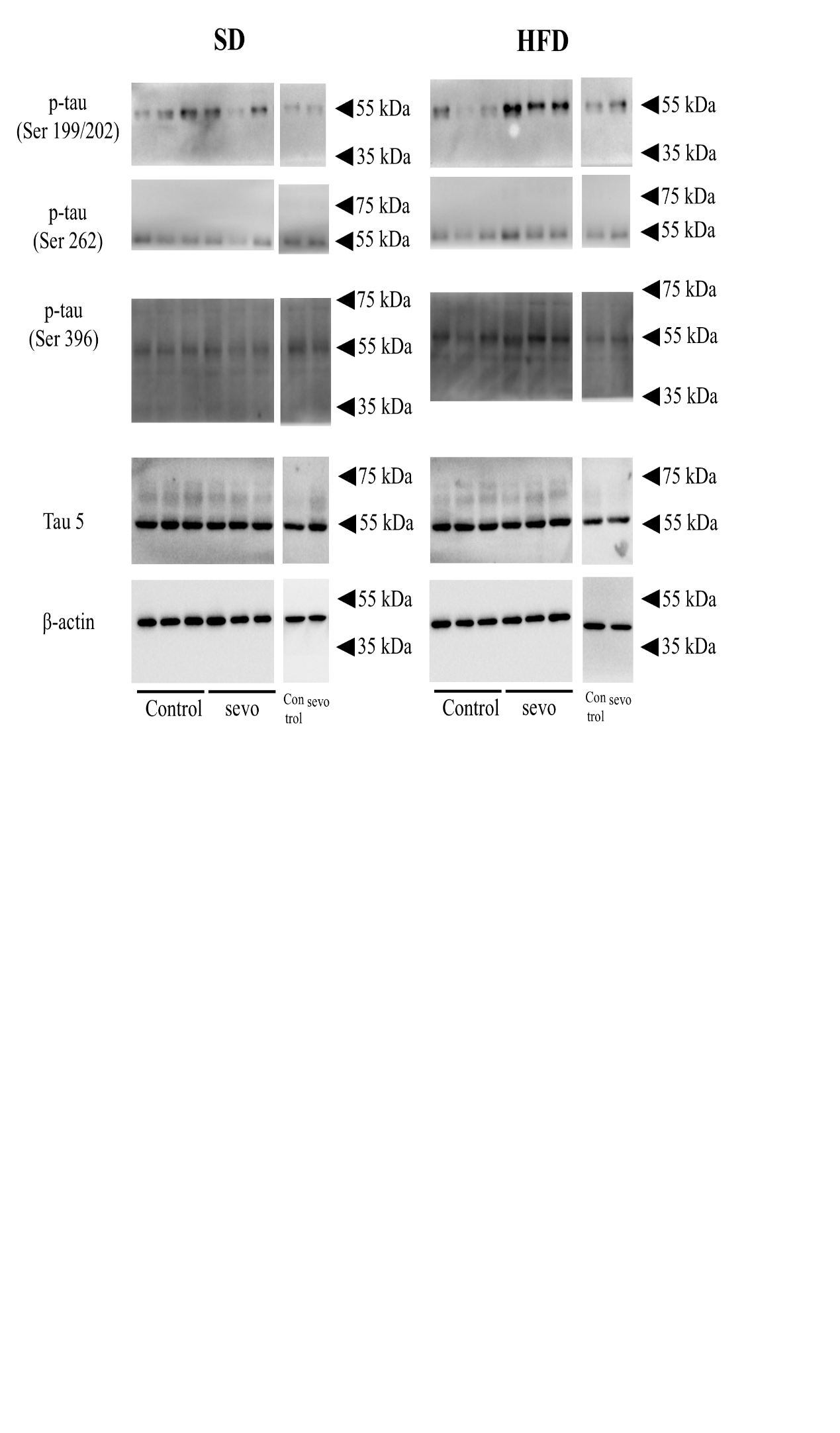
Fig. 3b Western blot


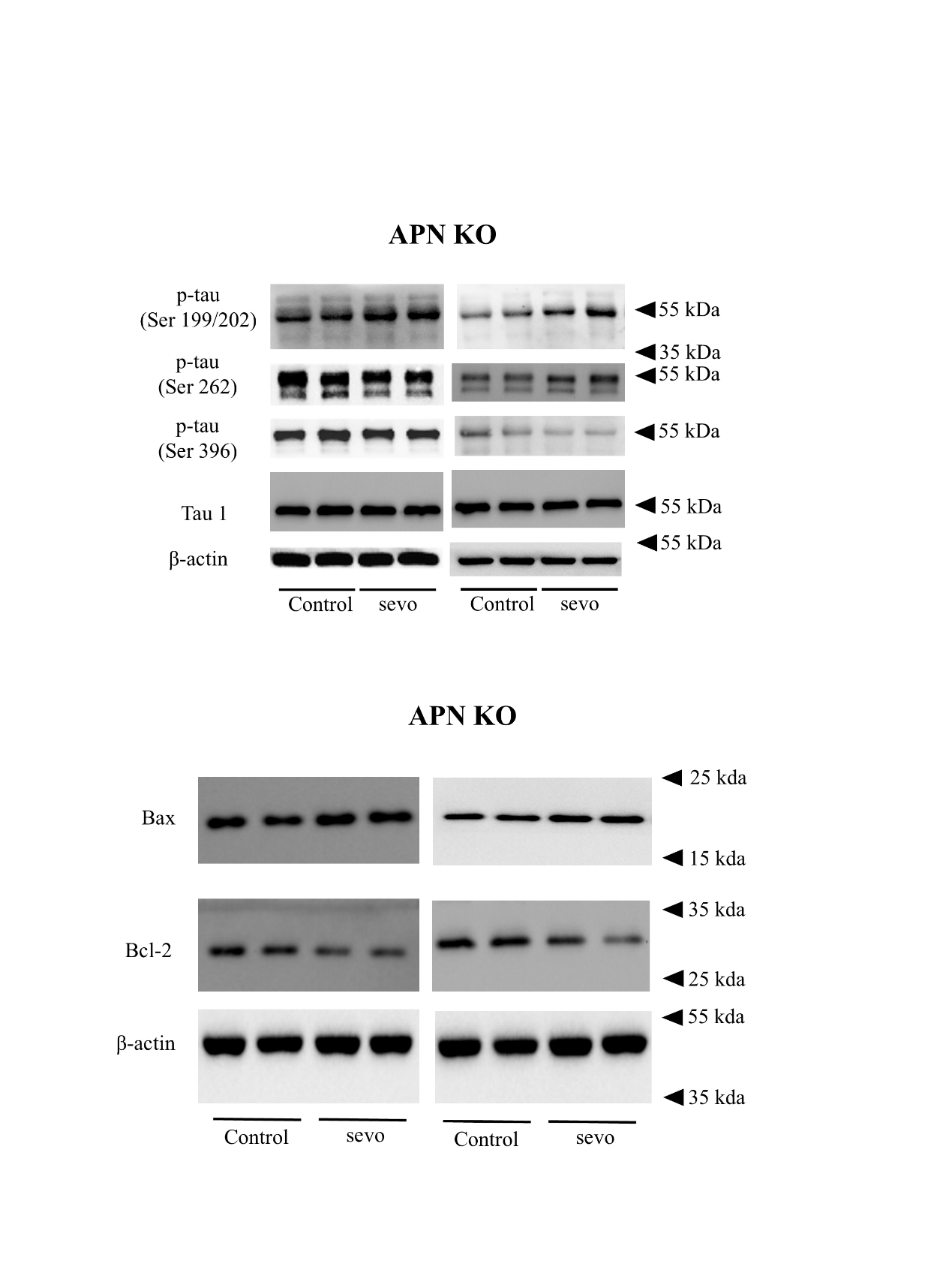
Fig. 4 Western blot


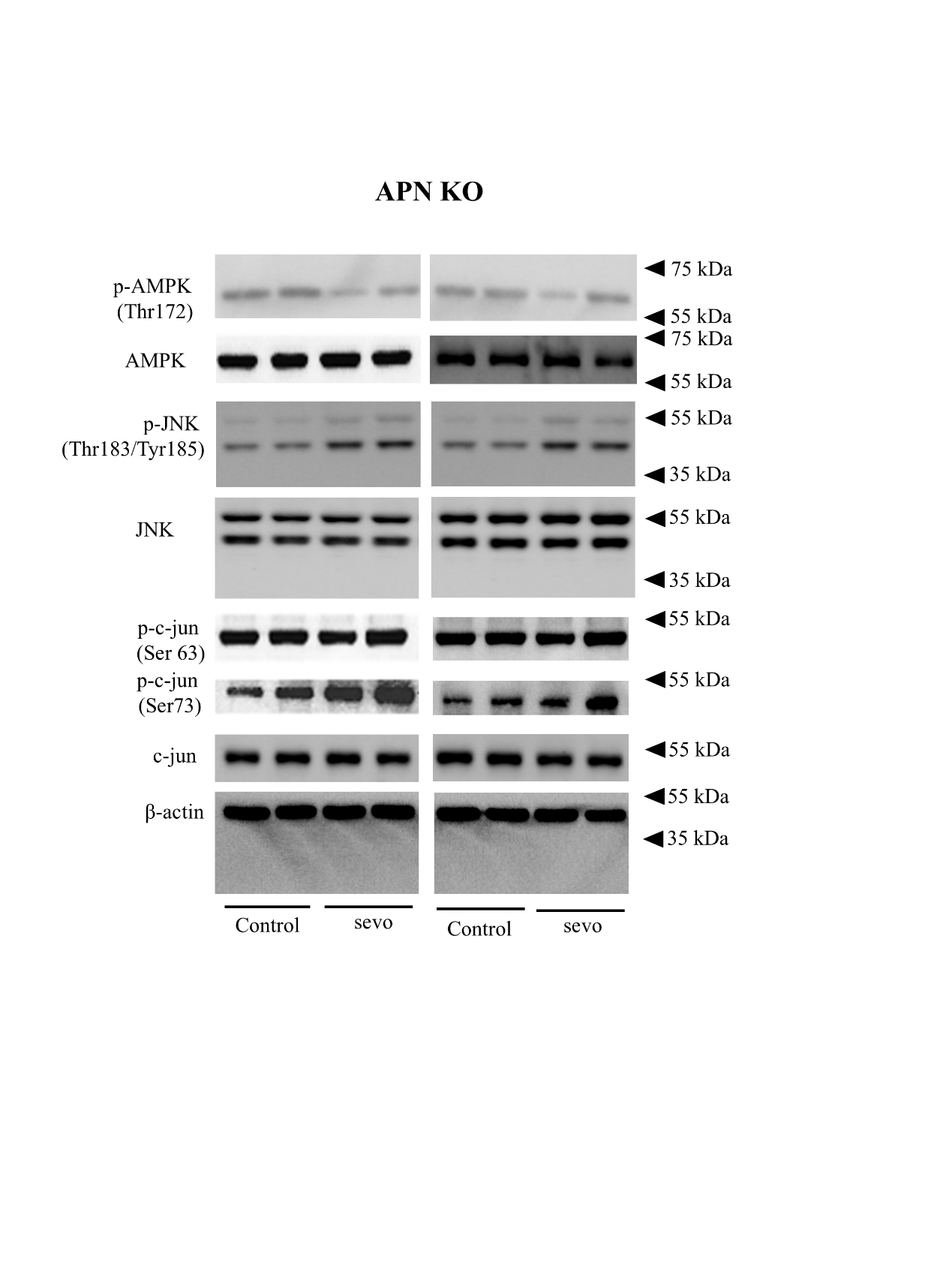
Fig. 5 Western blot

Fig. 6 Western blot
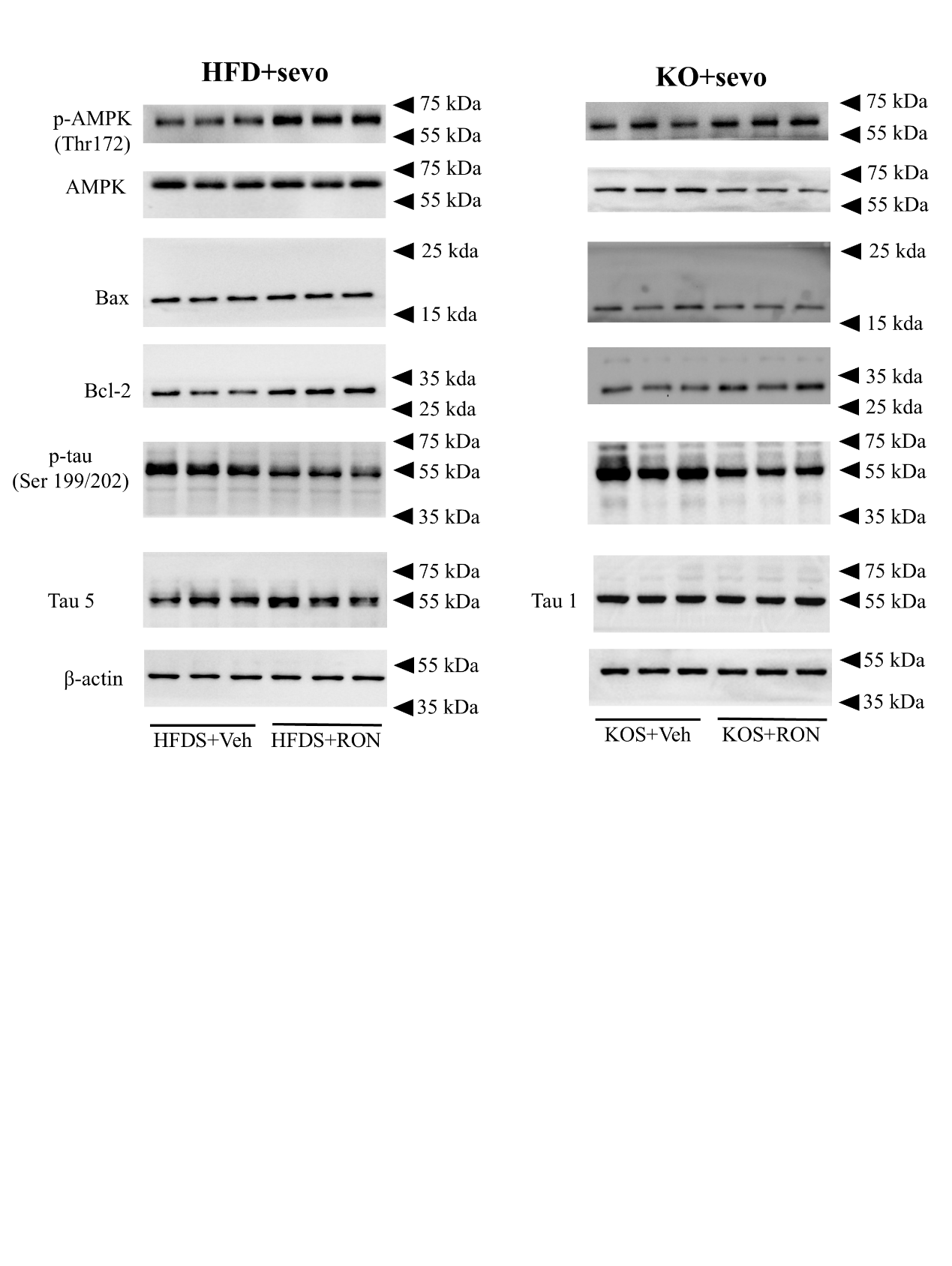

Supplement: Supplementary file 5 — Supplementary Material 5 [file 10020_2024_954_MOESM5_ESM.docx]
